# Sulfa Drugs as Inhibitors of Carbonic Anhydrase: New Targets for the Old Drugs

**DOI:** 10.1155/2014/162928

**Published:** 2014-09-08

**Authors:** Mariya al-Rashida, Sajad Hussain, Mehwish Hamayoun, Aisha Altaf, Jamshed Iqbal

**Affiliations:** ^1^Department of Chemistry, Forman Christian College (A Chartered University), Ferozepur Road, Lahore 54600, Pakistan; ^2^Centre for Advanced Drug Research, COMSATS Institute of Information Technology, Abbottabad 22060, Pakistan

## Abstract

Sulfa drugs are well-known antibacterial agents containing N-substituted sulfonamide group on para position of aniline ring (NH_2_RSO_2_NHR^′^). In this study 2,4-dichloro-1,3,5-triazine derivatives of sulfa drugs, sulfamerazine (**1b**), sulfaquinoxaline (**2b**), sulfadiazine (**3b**), sulfadimidine (**4b**), and sulfachloropyrazine (**5b**) (**1a**–**5a**) were synthesized and characterized. Their carbonic anhydrase inhibition activity was evaluated against bovine cytosolic carbonic anhydrase isozyme II (bCA II). For the sake of comparison the CA inhibition activity of the parent sulfa drugs (**1b**–**5b**) was also evaluated. A significant increase in CA inhibition activity of sulfa drugs was observed upon substitution with 2,4-dichloro-1,3,5-triazine moiety. Molecular docking studies were carried out to highlight binding site interactions. ADME properties were calculated to evaluate drug likeness of the compounds.

## 1. Introduction

Carbonic anhydrase (CA, EC 4.2.1.1) is a well-known ubiquitous zinc containing metalloenzyme that is found in animals including man, plants, bacteria, and archaea. The active site zinc ion (Zn^2+^) has been conserved in all classes of carbonic anhydrases [1]. This enzyme catalyzes an apparently simple yet physiologically important reaction of interconversion of water and carbon dioxide into bicarbonate and protons. Therefore CA has an important role to play in the transport of CO_2_ from metabolizing tissues to lungs. It is also responsible for maintaining acid/base and electrolyte balance in blood [2, 3]. Certain biosynthetic reactions are also assisted by CA such as lipogenesis, at the level of pyruvate carboxylation [4, 5], ureagenesis [6], and gluconeogenesis [6, 7]. In mammals carbonic anhydrase has sixteen different isozymes based on their distribution in tissues and subcellular localization. The cytosolic isozymes are CA I, CA II, CA III, CA VII, and CA XIII, whereas CA IV, CA IX, CA XII, CA XIV, and CA XV are membrane bound isozymes, and CA VA and CA VB are mitochondrial isozymes; CA VI is secreted isozyme mainly present in the saliva [8]. There are certain physiological disorders that are characterized by overexpression of CA [9–11], hence CAs have emerged as valuable drug targets. Many clinically established drugs are CA inhibitors and are used to treat disorders such as glaucoma, acidic ulcers, mountain/sea sickness, and epilepsy [12]. Carbonic anhydrase is also an important drug target for treating obesity and many sulfonamide inhibitors have proved to be efficient antiobesity agents [13–16]. The transmembrane isozymes CA IX and CA XII have been found to be overexpressed in hypoxic tumors (having acidic environment) whereas their distribution in normal cells remains low [17–22].

Sulfonamides and their derivatives are well-known inhibitors of carbonic anhydrase [18, 23]. Sulfa drugs are derived from sulfonamides; however all sulfonamides are not sulfa drugs, and the term sulfa drug is only used for clinically used antibacterial agents that are structurally derived from 4-aminobenzenesulfonamide, where the sulfonamide nitrogen is substituted (NH_2_RSO_2_NHR′) [24, 25]. The carbonic anhydrase inhibition activity of sulfa drugs has not been explored. Previously 1,3,5-triazine [26, 27] and 1,2,4-triazine [28] derivatives of different sulfonamides have been reported as efficient inhibitors of CA. Herein we report the synthesis of new 2,4,6-trichloro-1,3,5-triazine (TCT) derivatives of sulfa drugs (**1a**–**5a**) and their carbonic anhydrase inhibition activity against bovine cytosolic carbonic anhydrase II (bCA II). For the purpose of comparison, the carbonic anhydrase inhibition activity of parent sulfa drugs (**1b**–**5b**) is also reported.

## 2. Material and Methods

All chemicals used were purchased from either Sigma or Aldrich and used as such without further purification. Commercially available solvents were used. Ethanol was distilled and dried using standard methods and stored over molecular sieves. Reaction progress and product purity were checked via precoated TLC plates (silica gel, 0.2 mm, 60 HF_254_, Merck). TLC spots were visualized under short and long wavelength UV light. Bovine cytosolic carbonic anhydrase II (bCA II) was used. Melting points were taken on a Gallenkamp melting point apparatus and were uncorrected. FTIR spectra were taken on Perkin Elmer Spectrum BX-II. LECO CHNS 630 series elemental analyzer (model 630-200-200) was used for elemental analysis. For ^1^H and ^13^C-NMR analysis Bruker Avance DRX500 spectrometer was used with TMS as an internal standard and DMSO-d_6_ as solvent.

### 2.1. General Method of Synthesis

For the synthesis of TCT derived sulfa drugs, 2,4,6-trichloro-1,3,5-triazine (TCT, 0.01 mol), respective sulfa drug (0.01 mol), and sodium carbonate (0.1–0.2 g) were taken in a round bottom flask, 20 mL of ethanol and 5–7 mL acetone were added to it, and stirring was continued until a clear solution resulted. The reaction mixture was refluxed with constant stirring. After 2 hours solid precipitate began to appear in the reaction mixture; the reaction was allowed to continue for another hour after which the solid product was filtered, washed, and dried. Compounds were recrystallized with a mixture of acetone and acetonitrile.

#### 2.1.1. Synthesis of 4-[(4,6-Dichloro-1,3,5-triazin-2-yl)amino]-*N*-(4-methylpyrimidin-2-yl)benzenesulfonamide (**1a**)

Yield 80%; m.p. 235–237°C; IR (*ν*, cm^−1^): 3438 (NH_2_), 1145 (SO_2_^sym^), 1341 (SO_2_^asym^), 779 (C–Cl); Anal. calcd for C_14_H_11_Cl_2_N_7_O_2_S: C(40.79%), H(2.69%), N(23.78%), S(7.78%); found C(39.89%), H(3.4%), N(23.18%), S(7.54%). ^1^H-NMR (500 MHz, DMSO-d_6_), *δ* (ppm): 2.29 (s, 3H, –CH_3_), 8.31 (1H, m, H4′′), 7.99 (2H, d, ^3^ *J* = 10 Hz, H3′, H5′), 7.65 (2H, d, ^3^ *J* = 10 Hz, H2′, H6′), 7.78 (1H, d, ^3^ *J* = 5 Hz, H5′′), 11.13 (s, 1H, SO_2_NH), 8.34 (s, 1H, NH). ^13^C-NMR (125 MHz, DMSO-d_6_), *δ* (ppm): 157.70 (C1, C2), 156.99 (C3), 156.65 (C1′′), 150.02 (C1′), 130.08 (C3′′, C5′′), 129.11 (C3′, C5′), 120.74 (C4′), 119.59 (C2′, C6′), 114.85 (C4′′), 23.45 (CH_3_).

#### 2.1.2. Synthesis of 4-[(4,6-Dichloro-1,3,5-triazin-2-yl)amino]-*N*-(quinoxalin-2-yl)benzenesulfonamide (**2a**)

Yield 87%; m.p. 268–270°C; IR (*ν*, cm^−1^): 3248 (NH_2_), 1151 (SO_2_^sym^), 1314 (SO_2_^asym^), 727 (C–Cl); Anal. calcd for C_17_H_11_Cl_2_N_7_O_2_S: C (45.55%) H(2.47%), N (21.87%), S(7.15%); found C(44.87%), H(2.71%), N(20.90%), S(7.54%). ^1^H-NMR (500 MHz, DMSO-d_6_), *δ* (ppm): 8.08 (2H, d, ^3^ *J* = 10 Hz, H3′, H5′), 7.93 (2H, d, ^3^ *J* = 10 Hz, H2′, H6′), 7.79 (1H, s, H9′′), 7.78 (2H, m, H4′′, H7′′), 7.72 (2H, m, H5′′, H6′′), 10.82 (s, 1H, SO_2_NH), 8.63 (s, 1H, NH). ^13^C-NMR (125 MHz, DMSO-d_6_), *δ* (ppm): 164.08 (C3), 154.14 (C1, C2), 150.02 (C1′′), 146.19 (C1′), 142.16 (C3′′), 131.09 (C8′′), 129.12 (C3′, C5′), 128.87 (C4′′, C7′′), 127.41 (C5′′, C6′′), 120.91 (C4′), 120.06 (C2′, C6′).

#### 2.1.3. Synthesis of 4-[(4,6-Dichloro-1,3,5-triazin-2-yl)amino]-*N*-(pyrimidin-2-yl)benzenesulfonamide (**3a**)

Yield 88%; m.p. 221–223°C; IR (*ν*, cm^−1^): 3286 (NH_2_), 1143 (SO_2_^sym^), 1335 (SO_2_^asym^), 688 (C–Cl); Anal. calcd for C_13_H_9_Cl_2_N_7_O_2_S: C(39.21%), H(2.28%), N(24.62%), S(8.05%); found C(39.19%), H(2.46%), N(24.17%), S(8.51%). ^1^H-NMR (500 MHz, DMSO-d_6_), *δ* (ppm): 8.50 (2H, d, ^3^ *J* = 10 Hz, H3′, H5′), 7.96 (2H, d, ^3^ *J* = 10 Hz, H2′, H6′), 7.77 (2H, d, ^3^ *J* = 5 Hz, H3′′, H5′′), 7.04 (1H, t, ^3^ *J* = 5 Hz, H4′′), 8.0 (1H, s, NH), 10.82 (1H, s, SO_2_NH). ^13^C-NMR (125 MHz, DMSO-d_6_), *δ* (ppm): 158.48 (C1, C2), 157.03 (C3), 154.21 (C1′′), 141.81 (C1′), 134.89 (C3′′, C5′′), 128.92 (C3′, C5′), 120.81 (C4′), 120.07 (C2′, C6′), 115.91 (C4′′).

#### 2.1.4. Synthesis of 4-[(4,6-Dichloro-1,3,5-triazin-2-yl)amino]-N-(4,6-dimethylpyrimidin-2-yl)benzenesulfonamide (**4a**)

Yield 84%; m.p. 327–329°C; IR (*ν*, cm^−1^): 3211 (NH_2_), 1161 (SO_2_^sym^), 1334 (SO_2_^asym^), 697 (C–Cl); Anal. calcd for C_15_H_13_Cl_2_N_7_O_2_S: C(42.26%), H(3.07%), N(23.00%), S(7.52%); found C(42.87%), H(2.91%), N(23.20%), S(7.56). ^1^H-NMR (500 MHz, DMSO-d_6_), *δ* (ppm): 2.24 (6H, s, CH_3_), 6.74 (1H, s, H4′′), 7.95 (2H, d, ^3^ *J* = 10 Hz, H3′, H5′), 7.85 (2H, d, ^3^ *J* = 10 Hz, H2′, H6′), 7.83 (1H, s, NH), 10.61 (1H, s, SO_2_NH). ^13^C-NMR (125 MHz, DMSO-d_6_), *δ* (ppm): 23.02 (CH_3_), 164.0 (C1, C2), 156.33 (C3), 142.20 (C1′), 129.21 (C3′′, C5′′), 129.09 (C3′, C5′), 120.02 (C4′), 119.71 (C4′′).

#### 2.1.5. Synthesis of* N*-(6-Chloropyrazin-2-yl)-4-[(4,6-dichloro-1,3,5-triazin-2-yl)amino]benzenesulfonamide (**5a**)

Yield 85%; m.p. 236–238°C; IR (*ν*, cm^−1^): 3255 (NH_2_), 1158 (SO_2_^sym^), 1336 (SO_2_^asym^), 692 (C–Cl); Anal. calcd for C_13_H_8_Cl_3_N_7_O_2_S: C(36.09%), H(1.86%), N(22.66%), S(7.41%); found C(36.15%), H(1.94%), N(22.03%), S(7.44%). ^1^H-NMR (500 MHz, DMSO-d_6_), *δ* (ppm): 8.01 (2H, d, ^3^ *J* = 10 Hz, H3′, H5′), 7.62 (2H, d, ^3^ *J* = 10 Hz, H2′, H6′), 7.58 (1H, s, H4′′), 7.32 (1H, s, H6′′), 10.71 (s, 1H, SO_2_NH), 8.54 (s, 1H, NH). ^13^C-NMR (125 MHz, DMSO-d_6_), *δ* (ppm): 164.08 (C3), 154.14 (C1, C2), 150.02 (C1′′), 146.19 (C1′), 152.10 (C3′′), 129.12 (C3′, C5′), 132.87 (C4′′), 128.41 (C6′′), 121.21 (C4′), 120.13 (C2′, C6′).

### 2.2. *In Vitro* Carbonic Anhydrase Inhibition Assay

Carbonic anhydrase inhibition was measured by the reported method [29] after standardization of reaction conditions such as concentration of enzyme and substrate, buffer pH, and duration of reaction. The method is based on spectrophotometric determination* p*-nitrophenol, the CA catalyzed hydrolysis product of* p*-nitrophenyl acetate. The reaction mixture consisted of 60 *μ*L of 50 mM Tris-sulfate buffer (pH 7.6, containing 0.1 mM ZnCl_2_), 10 *μ*L (0.5 mM) test compound in 1% DMSO, and 10 *μ*L (50 U) bovine enzyme per well. Contents were mixed together and preincubated at 25°C for 10 minutes. Plates were preread at 348 nm using a 96-well plate reader. Substrate,* p*-nitrophenyl acetate (6 mM stock using <5% acetonitrile in buffer), 20 *μ*L was freshly prepared and added per well to achieve 0.6 mM concentration per well. Total reaction volume was made to 100 *μ*L. After incubating for 30 minute at 25°C, the contents were mixed and read at 348 nm. Suitable controls with DMSO and standard inhibitor acetazolamide (AZM) were included in the assay. Results reported are mean of three independent experiments (±SEM) and expressed as percent inhibitions calculated by the following formula: Inhibition (%) = [100 − (abs of test comp/abs of control) × 100)]. IC_50_ values of compounds (0.5 mM concentration) exhibiting >50% inhibition activity were calculated after suitable dilutions.

### 2.3. Molecular Docking Studies

#### 2.3.1. Receptor and Ligand Preparation

High resolution crystal structures of hCA II (0.90 Å) [30] and bCA II (1.95 Å) [31] were downloaded from the Protein Data Bank (PDB-IDs: 3K34 and 1V9E, resp.). Structural comparison and percent similarity (87.3%) and percent identity (80%) between bovine and human CA II have already been reported by our research group [32]. The RMSD with respect to the active site residues of the two proteins is 0.22 Å [32]. Method validation was done by redocking the bound ligand extracted from the active site of hCA-II (3K34). The docking method was able to reproduce the experimentally bound conformation of ligand in the active site with an RMSD of <2 Å. Method validation via self-docking could not be carried out for bCA II, since this enzyme did not contain any cocrystallized ligand. On this basis hCA-II was selected to carry out further molecular docking studies. For docking the receptor (hCA II) was prepared using DockPrep utility of Chimera software [33], which includes standard preparation steps such as adding hydrogen atoms and adding gasteiger charges using ANTECHAMBER [34] and repairing incomplete side chains (if any) using Dunbrack rotamer library. A charge of +2 was added on the zinc atom. Before docking the structures of all molecules were drawn using ACD/ChemSketch [35]. Gasteiger charges were added on each ligand using ANTECHAMBER [34] and the energy of each molecule was minimized through 100 steepest descents and 100 conjugate gradient steps using a step size of 0.02 each using Chimera [33]. Before docking, compounds were protonated (as in aqueous environment) using LeadIT 2.1.7 [36].

#### 2.3.2. Docking Methodology

For docking studies the binding site was defined, using LeadIT [36] with the aid of reference ligand which had cocrystallized with the enzyme. The binding site included amino acid residues within 8.0 Å of the active site. Docking studies were carried out using LeadIT [36] program that incorporates FlexX for docking calculations. The default docking and scoring parameters were kept for the docking calculations; first top 10 docked conformations of lowest energy were retained for detailed analysis of binding site interactions and binding modes. In order to validate the docking protocol, method validation was carried out by redocking the ligand extracted from 3K34; the docking was successfully able to reproduce the correct binding mode for the native ligand with an RMSD of <2.

## 3. Results and Discussion

### 3.1. Chemistry

**Figure 1:**
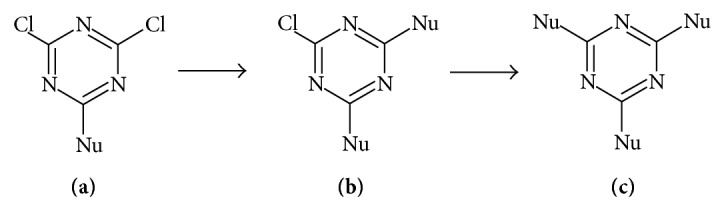
Decreasing order of ease of nucleophilic substitution on 2,4,6-trichloro-1,3,5-trizine ring, where Nu is a nucleophile [37].

2,4,6-Trichloro-1,3,5-triazine is an important and versatile molecule that provides the possibility of nucleophilic replacement at all three chlorine atoms. The synthetic utility and biological activities of this molecule are well known [37]. The ease of nucleophilic chloride replacement decreases as the number of chlorine atoms to be replaced decreases; thus nucleophilic replacement that results in synthesis of (**a**) proceeds far more swiftly than subsequent reactions that result in synthesis of molecules of types (**b**) and (**c**) (Figure 1).

**Scheme 1:**
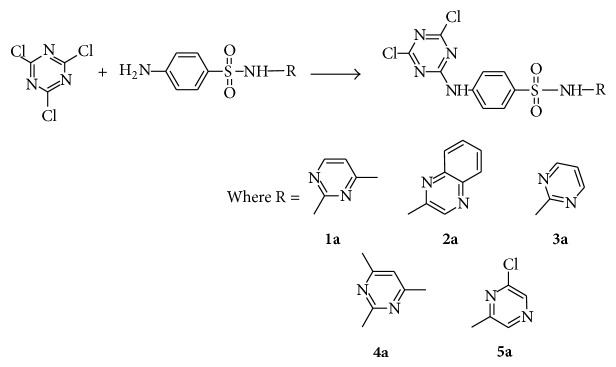
Synthesis of 2,4,6-trichloro-1,3,5-triazine (TCT) derivatives (**1a**–**5a**) of sulfa drugs sulfamerazine (**1b**), sulfaquinoxaline (**2b**), sulfadiazine (**3b**), sulfadimidine (**4b**), and sulfachloropyrazine (**5b**), respectively.

**Figure 2:**
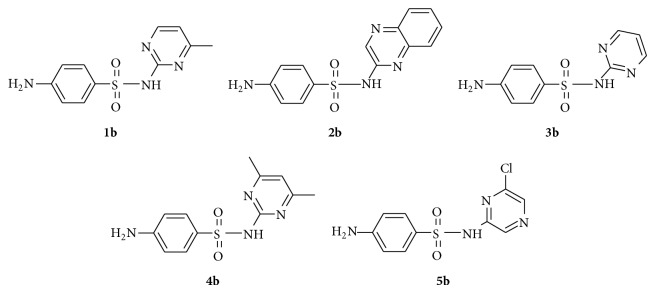
Structures of sulfa drugs, sulfamerazine (**1b**), sulfaquinoxaline (**2b**), sulfadiazine (**3b**), sulfadimidine (**4b**), and sulfachloropyrazine (**5b**).

Thus by reacting equimolar amounts of 2,4,6-trichloro-1,3,5-triazine and sulfa drugs, sulfamerazine (**1b**), sulfaquinoxaline (**2b**), sulfadiazine (**3b**), sulfadimidine (**4b**), and sulfachloropyrazine (**5b**), products of type (**a**) (see Scheme 1) were easily obtained without interference from di- or tri-substituted products. The structures of sulfa drugs used** 1b**–**5b** are given in Figure 2.

### 3.2. bCA II Inhibition Studies and SAR

The synthesized compounds (**1a**–**5a**) and their parent sulfa drugs (**1b**–**5b**) were screened against bovine cytosolic carbonic anhydrase II (bCA II). The CA active site residues involved in direct zinc binding are His94, His96, and His119. In addition the substrate (CO_2_) association pocket, Thr199 loop, and the histidine proton shuttle mechanism (for regeneration of active site Zn bound hydroxide ion) are essential for CA catalytic activity and are highly conserved in all alpha class CAs. For this reason initial screening against bCA-II was carried out. For comparison the bCA II inhibition activity of clinically used standard CA inhibitor acetazolamide (AZM) was also carried out (Table 1). The CA inhibition activity for all compounds is given in Table 1. All chloro triazine derived compounds were active inhibitors of bCA II. A significant increase in CA inhibition activity was observed for sulfa drugs containing chloro triazine moieties. Sulfa drugs (**1b**–**5b**) also exhibited the potential to inhibit bCA II; however their inhibitor potency is not significant. The most active sulfa drug was** 2b** showing CA inhibition up to 51.8% followed by** 5b** (42.4%). The corresponding chloro triazine derivative of** 5b**, that is, compound** 5a**, was the most active inhibitor of bCA II having IC_50_ value of 1.49 *μ*M. For compounds** 1a**–**5a**, the bCA II inhibition activity decreases in the order** 5a** >** 1a** >** 4a** >** 3a** >** 2a**, whereas for the parent sulfa drugs** 1b**–**5b**, the inhibition activity decreases as** 2b** >** 5b** >** 4b** >** 3b** >** 1b**.

**Figure 3:**
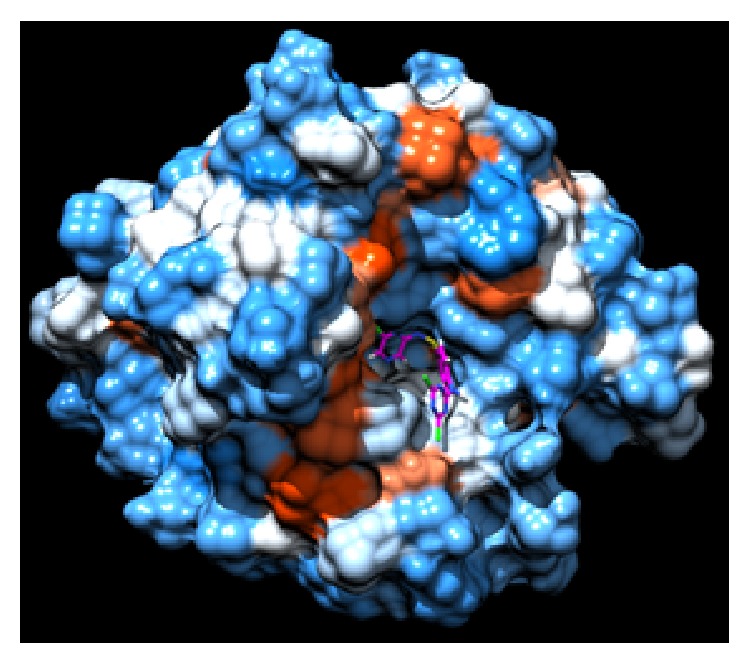
Compound** 5a** inside binding pocket of hCA II.** 5a** is shown in magenta color with sticks representation. The hydrophobic surfaces are drawn using Chimera and the color scheme is according to* Kyte-Doolittle coloring scheme;* blue color indicates most hydrophilic surface (hence polar residues), white for neutral, and orange for hydrophobic surface (nonpolar residues).

**Figure 4:**
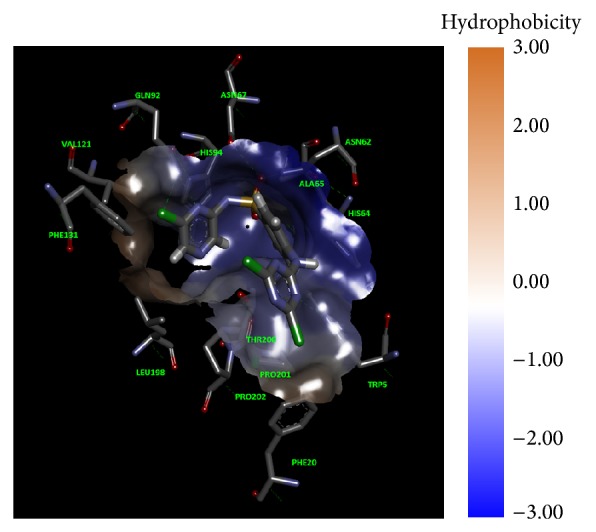
Interactions of compound** 5a** with active site residues.

**Figure 5:**
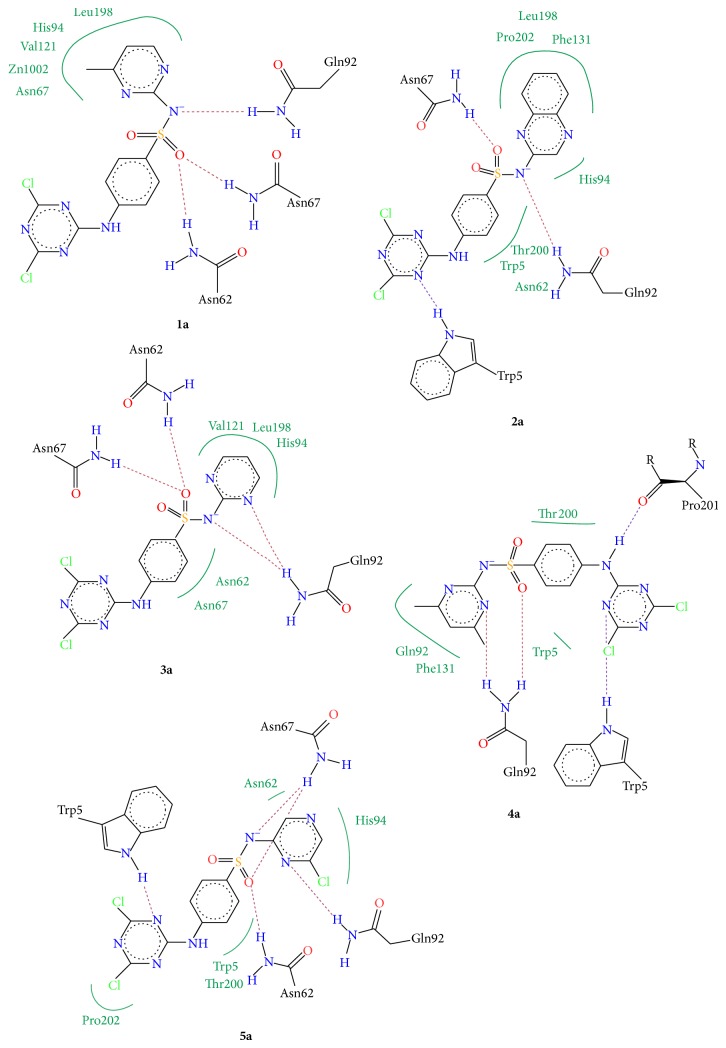
Interaction diagrams of the selected docked conformations for compounds** 1a**–**5a**. Hydrogen bond interactions are indicated with dotted lines and hydrophobic interactions are shown with green lines.

**Figure 6:**
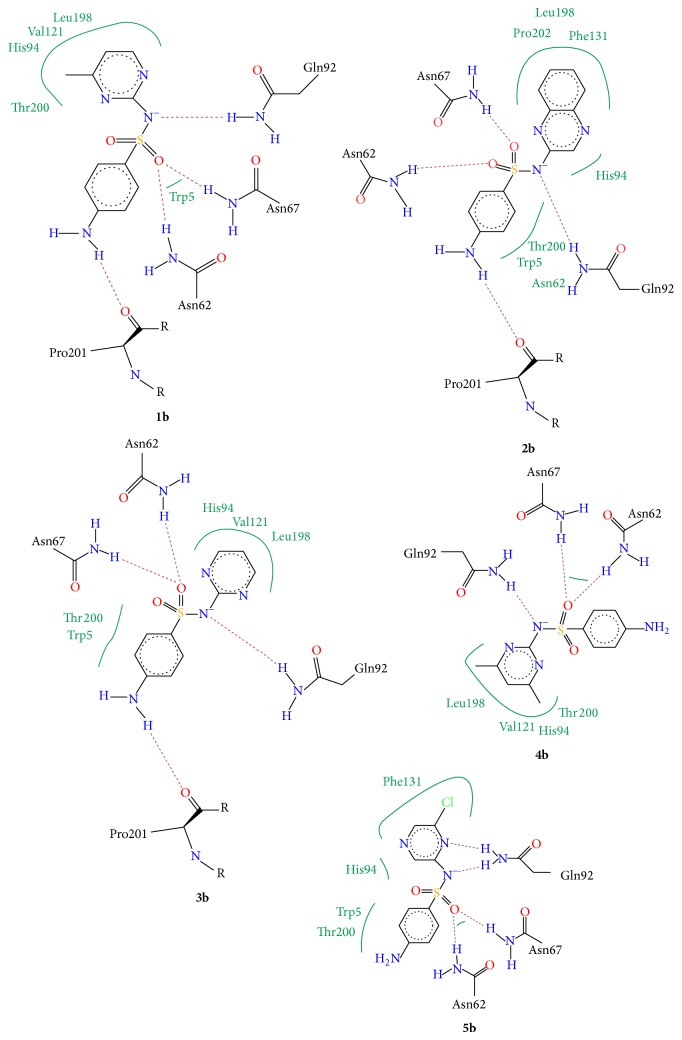
Interaction diagrams of the selected docked conformations for compounds** 1b**–**5b**. Hydrogen bond interactions are indicated with dotted lines and hydrophobic interactions are shown with green lines.

Molecular docking studies were carried out to investigate the mode of binding as well as important binding site interactions that could possibly explain the increased CA inhibition observed for chloro triazine derived compounds. Molecular docking studies were performed using LeadIT software. Before starting the molecular docking studies, the docking methodology was validated by redocking the ligand (that had cocrystallized with the hCA II enzyme 3K34). The docking method successfully reproduced the binding mode of native ligand with an RMSD of <2 Å. Although none of the compounds tested here contained a free sulfonamide group (SO_2_NH_2_), which is the typical zinc binding function that binds directly to the Zn^2+^ metal center of CA, still all compounds were able to inhibit this enzyme. Molecular docking studies reveal that, in addition to forming stable hydrogen bond interactions with surrounding amino acid residues as well as certain favorable hydrophobic interactions, these molecules were able to inhibit the enzyme by fitting inside the entrance of the active site such that the aryl group substituted on the sulfonamide nitrogen fits inside the hydrophobic pocket (the typical arylsulfonamide binding pocket) and the dichloro-triazine moiety fills the adjacent hydrophilic pocket (Figures 3 and 4). In all compounds studied, the sulfonamide group did not form a direct bond with the Zn^2+^ metallic centre, as expected, since the sulfonamide nitrogen atom is not free and is substituted. The binding modes of compounds were similar to one another. In general, for compounds** 1a**–**5a**, the sulfonamide nitrogen makes hydrogen bond interaction with Gln92 and sulfonamide oxygen makes hydrogen bond interactions with Asn62 and Asn67 (Figure 5). Similar interactions were observed for sulfonamide group of sulfa drugs** 1b**–**5b** (Figure 6). In most binding conformations for** 1a**–**5b**, the nitrogen atom of the triazine ring was found to be involved in hydrogen bond contact with Trp5. Common residues involved in hydrophobic and van der Waals interactions are Leu198, Val121, His94, Thr200, and Phe131. As indicated by molecular docking studies compounds** 1a**–**5a** are better able to inhibit CA II as compared to compounds** 1b**–**5b**, due to the presence of additional 2,4-dichloro-1,3,5-triazine moiety that fits in the adjacent hydrophilic pocket as seen in Figures 3 and 4.

Docking scores and their corresponding ranks are given in Table 2. The ranks of docked ligand conformations with most favorable binding mode were decided after running Hyde affinity assessment of first 10 lowest energy docked conformations. Hyde Affinity Assessment is a utility included in the LeadIT software; it allows selection of best docked conformation on the basis of calculated binding free energy. The docked conformation which has the least binding free energy (Δ*G*, KJ/mol) is most favorable. Thus, out of 10 lowest energy conformations, the conformation that had the lowest binding free energy Δ*G*, after Hyde assessment, was chosen and its corresponding rank and docking score are given in Table 2. The 2D interaction diagrams of binding site interactions are given in Figures 5 and 6.

In drug discovery process, the evaluation of ADME properties (Absorption, Distribution, Metabolism, and Excretion) is of considerable importance, since the likelihood of success of a molecule as a drug greatly depends on its favorable ADME. Accordingly the parameters selected for this study include octanol-water distribution coefficients (*S* + log⁡*P* and *M*log⁡*P*), the pH dependent octanol-water distribution coefficient (*S* + log⁡*D*), number of hydrogen bond donors (HBDH), hydrogen bond acceptor (sum of nitrogen and oxygen atoms MNO), and topological polar surface area (TPSA). The evaluation of TPSA is used as a model to assess the ability of compounds to cross the blood brain barrier (BBB). Molecules with TPSA < 60 Å^2^ are usually expected to be completely absorbed whereas molecules with TPSA > 140 Å^2^ are generally unwanted and cannot be expected to exhibit sufficient bioavailability [38]. Typically, compounds having molecular weight less than 500, number of hydrogen bond acceptor (HBDH) less than 10, number of hydrogen bond donor (MNO) less than 5, and a log⁡*P* value of less than 5 are considered to be orally bioavailable with favorable ADME profile [39, 40]. The calculated ADME properties of compounds** 1a**–**5a** and** 1b**–**5b** are given in Table 3. All synthesized compounds showed favorable ADME properties. No violation of Lipinski's rule of 5 was observed.

## 4. Conclusions

Sulfa drugs are well known antibacterial agents containing N-substituted sulfonamide group on para position of aniline ring (NH_2_RSO_2_NHR′). In this study 2,4-dichloro-1,3,5-triazine derivatives of sulfa drugs (sulfamerazine, sulfaquinoxaline, sulfadiazine, sulfadimidine, and sulfachloropyrazine) (**1a**–**5a**) were synthesized and characterized. Their carbonic anhydrase inhibition activity was evaluated against bCA II isozyme. For the sake of comparison the CA inhibition activity of parent sulfa drugs (**1b**–**5b**) was also evaluated. It is interesting to note here that although all compounds including sulfa drugs contained an N-substituted sulfonamide group, they were still able to inhibit CA activity. Sulfa drugs exhibited weak CA inhibition (19.6–51.8%). However 2,4-dichloro-1,3,5-triazine derivatives of sulfa drugs (**1a**–**5a**) had significantly improved CAI activity (IC_50_ = 1.49–24.9 *μ*M) and only compound** 2a** had IC_50_ value of 105.3 *μ*M. For compounds** 1a**-**5a**, the bCA II inhibition activity decreases in the order** 5a** >** 1a** >** 4a** >** 3a** >** 2a**, whereas for the parent sulfa drugs** 1b**–**5b**, the inhibition activity decreases as** 2b** >** 5b** >** 4b** >** 3b** >** 1b**. Molecular docking studies were carried out to give insights into the binding site interactions. ADME properties were calculated to evaluate the drug likeness of synthesized compounds. All compounds indicated a favorable ADME profile.

## Figures and Tables

**Table 1:** bCA II inhibition data for compounds **1a**–**5a** and their parent compounds **1b**–**5b**.

Compounds	IC_50_ ± SEM (*µ*M) (or % inhibition)^a^
**1a **	4.14 ± 0.02
**2a **	105.3 ± 0.16
**3a **	24.9 ± 0.03
**4a **	14.9 ± 0.02
**5a **	1.49 ± 0.006
**1b**	(19.6 ± 0.2)
**2b**	(51.8 ± 0.6)
**3b**	(31.2 ± 0.5)
**4b**	(41.7 ± 1.2)
**5b**	(42.4 ± 0.7)
AZM	1.16 ± 0.02

^a^% inhibition at 0.5 mM concentration of tested compounds; AZM: acetazolamide.

**Table 2:** Docking scores and their corresponding ranks as judged by Hyde affinity assessment.

Compound	Docking score	Rank	Δ*G* (KJ/mol)
**1a**	−15.96	7	−6
**2a**	−19.87	1	−2
**3a**	−15.04	9	−5
**4a**	−16.64	9	−1
**5a**	−17.06	6	−15
**1b**	−20.37	7	−8
**2b**	−20.37	7	−8
**3b**	−20.66	6	−7
**4b**	−18.18	7	−11
**5b**	−15.55	8	−5

**Table 3:** Calculated ADME properties of compounds **1a**–**5a** and **1b**–**5b**.

Compound	*S* + log⁡*P*	*S* + log⁡*D*	*M*log⁡*P*	MWt	HBDH	MNO	TPSA
**1a**	2.712	1.523	1.93	412.259	2	9	122.65
**2a**	3.346	2.077	1.69	448.292	2	9	122.65
**3a**	2.397	0.965	1.681	398.232	2	9	122.65
**4a**	2.997	2.08	2.173	426.286	2	9	122.65
**5a**	2.766	0.588	1.118	432.677	2	9	122.65
**1b**	0.542	−0.112	0.779	264.307	3	6	97.97
**2b**	1.168	0.676	0.633	300.341	3	6	97.97
**3b**	0.169	−0.681	0.487	250.28	3	6	97.97
**4b**	0.926	0.484	1.06	278.334	3	6	97.97
**5b**	0.811	−0.468	0.235	284.725	3	6	97.97

## References

[1] Tripp B. C., Smith K., Ferry J. G. (2001). Carbonic anhydrase: new insights for an ancient enzyme. *The Journal of Biological Chemistry*.

[2] Breton S. (2001). The cellular physiology of carbonic anhydrases. *Journal of the Pancreas*.

[3] Henry R. P. (1996). Multiple roles of carbonic anhydrase in cellular transport and metabolism. *Annual Review of Physiology*.

[4] Lynch C. J., Fox H., Hazen S. A., Stanley B. A., Dodgson S., Lanoue K. F. (1995). Role of hepatic carbonic anhydrase in de novo lipogenesis. *Biochemical Journal*.

[5] Hazen S. A., Waheed A., Sly W. S., LaNoue K. F., Lynch C. J. (1996). Differentiation-dependent expression of CA V and the role of carbonic anhydrase isozymes in pyruvate carboxylation in adipocytes. *The FASEB Journal*.

[6] Shah G. N., Rubbelke T. S., Hendin J., Nguyen H., Waheed A., Shoemaker J. D., Sly W. S. (2013). Targeted mutagenesis of mitochondrial carbonic anhydrases VA and VB implicates both enzymes in ammonia detoxification and glucose metabolism. *Proceedings of the National Academy of Sciences of the United States of America*.

[7] Dodgson S. J., Cherian K. (1989). Mitochondrial carbonic anhydrase is involved in rat renal glucose synthesis. *American Journal of Physiology—Endocrinology and Metabolism*.

[8] Alterio V., di Fiore A., D'Ambrosio K., Supuran C. T., de Simone G. (2012). Multiple binding modes of inhibitors to carbonic anhydrases: how to design specific drugs targeting 15 different isoforms?. *Chemical Reviews*.

[9] Kirkpatrick J. P., Rabbani Z. N., Bentley R. C., Hardee M. E., Karol S., Meyer J., Oosterwijk E., Havrilesky L., Secord A. A., Vujaskovic Z., Dewhirst M. W., Jones E. L. (2008). Elevated CAIX expression is associated with an increased risk of distant failure in early-stage cervical cancer. *Biomarker Insights*.

[10] Dorai T., Sawczuk I. S., Pastorek J., Wiernik P. H., Dutcher J. P. (2005). The role of carbonic anhydrase IX overexpression in kidney cancer. *European Journal of Cancer*.

[11] Scozzafava A., Supuran C. T. (2014). Glaucoma and the applications of carbonic anhydrase inhibitors. *Subcellular Biochemistry*.

[12] Supuran C. T., Scozzafava A. (2007). Carbonic anhydrases as targets for medicinal chemistry. *Bioorganic & Medicinal Chemistry*.

[13] Supuran C. T., di Fiore A., de Simone G. (2008). Carbonic anhydrase inhibitors as emerging drugs for the treatment of obesity. *Expert Opinion on Emerging Drugs*.

[14] Scozzafava A., Supuran C. T., Carta F. (2013). Antiobesity carbonic anhydrase inhibitors: a literature and patent review. *Expert Opinion on Therapeutic Patents*.

[15] de Simone G., Supuran C. T. (2007). Antiobesity carbonic anhydrase inhibitors. *Current Topics in Medicinal Chemistry*.

[16] de Simone G., Dio Fiore A., Supuran C. T. (2008). Are carbonic anhydrase inhibitors suitable for obtaining antiobesity drugs?. *Current Pharmaceutical Design*.

[17] Supuran C. T. (2012). Inhibition of bacterial carbonic anhydrases and zinc proteases: from orphan targets to innovative new antibiotic drugs. *Current Medicinal Chemistry*.

[18] Supuran C. T., Scozzafava A. (2000). Carbonic anhydrase inhibitors: aromatic sulfonamides and disulfonamides act as efficient tumor growth inhibitors. *Journal of Enzyme Inhibition*.

[19] Pastorekova S., Zatovicova M., Pastorek J. (2008). Cancer-associated carbonic anhydrases and their inhibition. *Current Pharmaceutical Design*.

[20] Thiry A., Dogné J.-M., Masereel B., Supuran C. T. (2006). Targeting tumor-associated carbonic anhydrase IX in cancer therapy. *Trends in Pharmacological Sciences*.

[21] Guler O. O., de Simone G., Supuran C. T. (2010). Drug design studies of the novel antitumor targets carbonic anhydrase IX and XII. *Current Medicinal Chemistry*.

[22] Winum J.-Y., Rami M., Scozzafava A., Montero J.-L., Supuran C. (2008). Carbonic anhydrase IX: a new druggable target for the design of antitumor agents. *Medicinal Research Reviews*.

[23] Supuran C. T., Briganti F., Tilli S., Chegwidden W. R., Scozzafava A. (2001). Carbonic anhydrase inhibitors: sulfonamides as antitumor agents?. *Bioorganic and Medicinal Chemistry*.

[24] Brackett C. C., Singh H., Block J. H. (2004). Likelihood and mechanisms of cross-allergenicity between sulfonamide antibiotics and other drugs containing a sulfonamide functional group. *Pharmacotherapy*.

[25] Knowles S., Shapiro L., Shear N. H. (2001). Should celecoxib be contraindicated in patients who are allergic to sulfonamides? Revisiting the meaning of “sulfa– allergy. *Drug Safety*.

[26] Garaj V., Puccetti L., Fasolis G., Winum J.-Y., Montero J.-L., Scozzafava A., Vullo D., Innocenti A., Supuran C. T. (2005). Carbonic anhydrase inhibitors: novel sulfonamides incorporating 1,3,5-triazine moieties as inhibitors of the cytosolic and tumour-associated carbonic anhydrase isozymes I, II and IX. *Bioorganic and Medicinal Chemistry Letters*.

[27] Carta F., Garaj V., Maresca A., Wagner J., Avvaru B. S., Robbins A. H., Scozzafava A., McKenna R., Supuran C. T. (2011). Sulfonamides incorporating 1,3,5-triazine moieties selectively and potently inhibit carbonic anhydrase transmembrane isoforms IX, XII and XIV over cytosolic isoforms i and II: solution and X-ray crystallographic studies. *Bioorganic and Medicinal Chemistry*.

[28] Garaj V., Puccetti L., Fasolis G., Winum J.-Y., Montero J.-L., Scozzafava A., Vullo D., Innocenti A., Supuran C. T. (2004). Carbonic anhydrase inhibitors: synthesis and inhibition of cytosolic/tumor-associated carbonic anhydrase isozymes I, II, and IX with sulfonamides incorporating 1,2,4-triazine moieties. *Bioorganic & Medicinal Chemistry Letters*.

[29] Pocker Y., Stone J. T. (1967). The catalytic versatility of erythrocyte carbonic anhydrase. III. Kinetic studies of the enzyme-catalyzed hydrolysis of p-nitrophenyl acetate. *Biochemistry*.

[30] Behnke C. A., Le Trong I., Godden J. W., Merritt E. A., Teller D. C., Bajorath J., Stenkamp R. E. (2010). Atomic resolution studies of carbonic anhydrase II. *Acta Crystallographica Section D: Biological Crystallography*.

[31] Saito R., Sato T., Ikai A., Tanaka N. (2004). Structure of bovine carbonic anhydrase II at 1.95 Å resolution. *Acta Crystallographica Section D*.

[32] Ul-Haq Z., Usmani S., Mahmood U., Al-Rashida M., Abbas G. (2013). In-silico analysis of chromone containing sulfonamide derivatives as human carbonic anhydrase inhibitors. *Medicinal Chemistry*.

[33] Pettersen E. F., Goddard T. D., Huang C. C., Couch G. S., Greenblatt D. M., Meng E. C., Ferrin T. E. (2004). UCSF Chimera—a visualization system for exploratory research and analysis. *Journal of Computational Chemistry*.

[34] Wang J., Wang W., Kollman P. A., Case D. A. (2006). Automatic atom type and bond type perception in molecular mechanical calculations. *Journal of Molecular Graphics and Modelling*.

[35] ACD/ChemSketch http://www.acdlabs.com/.

[36] http://www.biosolveit.de/LeadIT/.

[37] Afonso C. A. M., Lourenço N. M. T., de Rosatella A. A. (2006). Synthesis of 2,4,6-tri-substituted-1,3,5-triazines. *Molecules*.

[38] Zakeri-Milani P., Tajerzadeh H., Islambolchilar Z., Barzegar S., Valizadeh H. (2006). The relation between molecular properties of drugs and their transport across the intestinal membrane. *Daru*.

[39] Asokkumar K., Prathyusha L. T., Umamaheshwari M., Sivashanmugam T., Subhadradevi V., Jagannath P., Madeswaran A., Salesheir F. (2012). Design, ADMET and docking studies on some novel chalcone derivatives as soluble epoxide hydrolase enzyme inhibitors. *Journal of the Chilean Chemical Society*.

[40] Clark D. E., Pickett S. D. (2000). Computational methods for the prediction of “drug-likeness”. *Drug Discovery Today*.

